# CD4^+^ lymphocyte adenosine triphosphate - a new marker in sepsis with acute kidney injury?

**DOI:** 10.1186/1471-2369-15-203

**Published:** 2014-12-18

**Authors:** Daniel Patschan, Malte Heeg, Maria Brier, Gunnar Brandhorst, Simon Schneider, Gerhard A Müller, Michael J Koziolek

**Affiliations:** Department of Nephrology & Rheumatology, Georg-August-University Göttingen, Göttingen, Germany; Department of Clinical Chemistry, Georg-August-University Göttingen, Göttingen, Germany; Department of Medical Statistics, Georg-August-University Göttingen, Göttingen, Germany; University Hospital of Göttingen, Robert-Koch-Straße 40, 37075 Göttingen, Germany; Department of Nephrology and Rheumatology, University Hospital of Göttingen, Robert-Koch-Straße 40, 37075 Göttingen, Germany

**Keywords:** ATP_CD4, Sepsis, AKI, Mortality, Kidney regeneration

## Abstract

**Background:**

AKI frequently develops in sepsis patients, significantly decreasing the overall prognosis. There are currently no diagnostic markers available which reliably predict the prognosis of sepsis-associated AKI. Recently, ATP content of CD4+ T cells (ATP_CD4) has been shown to correlate with survival in sepsis. The aim of the study was to determine ATP_CD4 in sepsis-associated AKI.

**Methods:**

Thirty-three patients with sepsis were prospectively analyzed for ATP_CD4 at three different time points. Results were related to survival, renal recovery, and further clinical/laboratory findings.

**Results:**

ATP_CD4 tended to lower in concentration at 48 h after onset of sepsis in those patients with complete renal recovery. There were no differences between patients with no AKI and those with AKI of different severity (AKIN 1-3). Urinary NGAL did not correlate with renal prognosis.

**Conclusion:**

ATP_CD4 may serve as risk predictor in sepsis-associated AKI. Lower concentrations may indicate a higher chance of complete renal recovery in sepsis.

## Background

Acute kidney injury (AKI) is still a frequent and serious complication in hospitalized patients with an incidence of 5-35% [1]. The most common cause is transient renal hypoperfusion with tubular cell dysfunction and, in more severe cases tubular cell apoptosis/necrosis. Therapeutic measures include elimination of the respective cause, stabilization of intravascular fluid volume, avoidance of nephrotoxic drugs, and treatment of secondary complications such as hyperkalemia and acidosis. Current diagnostic markers still do not allow an early diagnosis of AKI which partly accounts for the poor prognosis of the disease. In addition, *predicting* both, renal and general outcome in AKI remains difficult if not impossible. A promising candidate is the Neutrophil Gelatinase-Associated Lipocalin (NGAL) which can being used for predicting the need for renal replacement therapy but not mortality in AKI [2, 3]. Lately, the urinary IL-18/creatinine ratio has been shown to predict persistently elevated serum creatinine levels of ≥0.3 mg/dl after 6 months as compared to the baseline [4]. Nevertheless, all available diagnostic parameters are of limited value since they do not allow monitoring of tissue repair processes *per se*. Postischemic renal regeneration critically depends on a balanced interaction between pro- and anti-inflammatory cell populations. Among those are T and B cell, monocytes/macrophages, dendritic cells, granulocytes, and plasma cells [5]. However, a diagnostic method for analyzing activities of cells involved in renal tissue repair is missing yet. A pilot study on monitoring activity of CD4+ T cells in sepsis was published in 2010. This investigation revealed an association between low CD4+ ATP (ATP_CD4) content at the time of ICU admission and poor clinical outcome [6]. Although such phenomenon is far from being understood mechanistically, CD4+ ATP contents most likely reflect activity/competence of a certain population of immunocompetent cells. Since AKI is a frequent and deleterious complication in sepsis, we aimed to investigate ATP_CD4 in septic patients with versus without AKI. Our principal goal was to evaluate the prognostic power of ATP_CD4 in terms of kidney regeneration after sepsis-associated AKI.

## Methods

### Patients

All patients included in this prospective study were recruited from the intermediate care unit, or the intensive care unit of the department of Nephrology and Rheumatology (University Hospital Göttingen, Germany). The study protocol was approved after review by the medical local ethics committee of the University Hospital of Göttingen (“Klinisches Ethikkomitee der Georg-August-Universität Göttingen”). It was the only committee which approved the study since it was a single center analysis. The investigation conformed to the principles outlined in the Declaration of Helsinki and written informed consent was obtained from each subject. Acute kidney injury was defined using the AKIN criteria [7]. Patients with pre-existing CKD (chronic kidney disease) were also included into the study. In these patients, any further acute aggravation of renal dysfunction defined AKI if the AKIN criteria were applicable and/or if, for other reasons, dialysis treatment was initiated. Indications for dialysis were the presence of two or more of the following criteria: refractory hyperkalemia, increases of serum creatinine >3 mg/dl and/or of blood urea nitrogen >100 mg/dl at any given time point, and signs/symptoms of fluid overload due to diminished urine output, respectively. As in earlier studies [8, 9], sepsis was defined as systemic inflammatory response syndrome (SIRS) of infectious origin [10]. Thus, beside fulfilling the criteria of SIRS [11], almost all patients showed at least once, positive blood cultures for either Gram-positive or Gram-negative bacteria, and/or clinical symptoms of an infectious disease. For further clinical characterization a number of different parameters were documented on a daily basis. All data analyzed in this study were extracted from a database, belonging to the department of Nephrology & Rheumatology of the University Hospital of Göttingen. All patients were evaluated clinically and by laboratory findings at the following time points: 24, 48, and 96 hours after inclusion into the study. Complete renal recovery was defined as normalization of eGFR, partial renal recovery was defined as persistent eGFR decline without need for renal replacement therapy. The estimated GFR (eGFR) was calculated according to the method by Levey and colleagues [12] (Levey AS, Bosch JP, Lewis JB, et al; A more accurate method to estimate glomerular filtration rate from serum creatinine: a new prediction equation. Modification of Diet in Renal Disease Study Group. Ann Intern Med. 1999 Mar 16;130(6):461-70.).

### Quantification of ATP in CD4^+^ T cells

Quantification of ATP in CD4+ T cells was performed according to a previously published protocol using the Immuknow assay (Cylex Inc., Columbia, MD, USA) [6].

### Quantification of urinary NGAL

Urinary NGAL was measured using the microparticle immunoassay ‘ARCHITECT Urine NGAL’ (Abbott GmbH & Co. KG, Wiesbaden) according to the manufacturer’s protocol.

### Statistical analysis

All results are expressed mean ± SD. Differences between 3 or more groups were analyzed by ANOVA. Significance was considered at p < 0.05. Power analysis was performed using the programme G*Power®. The test being used was the f test (IDRE RESEARCH TECHNOLOGY GROUP).

## Results

### Patients

Patients’ characteristics are summarized in Table 1. A total of 33 patients were included into the study; 20 male, 13 female. The mean age of all patients was 66 ± 16 years. Sepsis occurred as a complication of the following diseases: pneumonia [13], urinary tract infection (3), pancreatitis (1), cholecystitis (1), and unknown primary infection [13]. During the course of the disease, 11 patients did not develop AKI while 22 presented acute renal dysfunction according to the respective criteria (AKIN 1 8 patients, AKIN 2 4 patients, AKIN 3 10 patients). Differences between the four groups were close to the level of significance (p = 0.054). The following categories were significantly different between the four groups: SAPS at admission (no AKI 28.09 ± 7.89, AKIN 1 38.71 ± 11.21, AKIN 2 42 ± 7.01, AKIN 40.6 ± 11.18, p = 0.016), positive microbiology (no AKI 4/11, AKIN 1 0/8, AKIN 2 4/4, AKIN 3 6/10, p < 0.01), eGFR at discharge (no AKI 118 ± 72 ml/min, AKIN 1 39 ± 12 ml/min, AKIN 2 41 ± 19 ml/min, AKIN 3 34 ± 31 ml/min, p < 0.01), and complete renal recovery (no AKI 11/11, AKIN 1 1/8, AKIN 2 1/4, AKIN 3 1/10, p < 0.001). All other categories did not significantly differ between the four groups (Body Mass Index, maximum SAPS, need for vasopressor therapy, survival, comorbidities such as liver failure, diabetes, hypertension, preexisting CKD, dialysis dependency at discharge).

**Table 1:** **Patients characteristics**

	Total	no AKI	AKIN 1	AKIN 2	AKIN 3	p-value
**n**	33	11	8	4	10	
**Age**	66.15 (±16.42)	60.09 (±16.77)	74 (±12.98)	80.25 (±6.75)	60.9 (±16.79)	0.054 n.s.
**Gender**						
male	20/33 (%)	6/11	5/8	2/4	7/10	0.86
female	13/33	5/11	3/8	2/4	3/10	n.s.
**BMI**	30.05 (±7.5)	28.43 (±7.89)	31.11 (±9.93)	26.03 (±1.38)	32.36 (±6.57)	0.467 n.s.
**SAPS at admission**	36.06 (±10.77)	28.09 (±6.89)	38.71 (±11.21)	42 (±7.07)	40.6 (±11.18)	0.016
**SAPS max**	39.32 (±10.65)	32.73 (±9.45)	42 (±7.75)	43.25 (±6.5)	43.4 (±12.17)	0.077 n.s.
**Microbiology positiv**	11/33	4/11	0/8	4/4	6/10	<0.01
**Vasopressor therapy**	11/33	3/11	4/8	0/4	4/10	0.33
**Survivor**	27/33	11/11	7/8	3/4	6/10	0.11
**Comorbidities**						
liver failure	3/33	0/11	0/8	0/4	3/10	0.085
diabetes mellitus	11/33	2/11	4/8	1/4	5/10	0.39
hypertension	13/33	2/11	5/8	1/4	5/10	
CKD	8/33	0/11	3/8	1/4	4/10	0.077
**baseline eGFR (ml/min/1.73 m** ^**2**^	73.29 ± 47.01	118.66 ± 27.5	39.73 ± 11.9	40.77 ± 19.37	34.8 ± 31.16	<0.01
**Discharge**						
eGFR	73.29 (±47.01)	118.66 (±27.5)	39.73 (±11.9)	40.77 (±19.37)	34.8 (±31.16)	<0.01
death	6/33	0/11	1/8	1/4	4/10	
dialysis dependency	1/33	0/11	0/8	0/4	1/10	
complete renal recovery	14/33	11/11	1/8	1/4	1/10	<0.001
partial renal recovery	12/33	0/11	6/8	2/4	4/10	

For further explanation see text.

### Primary study endpoint: ATP content in CD4^+^ T cells

**Figure 1:**
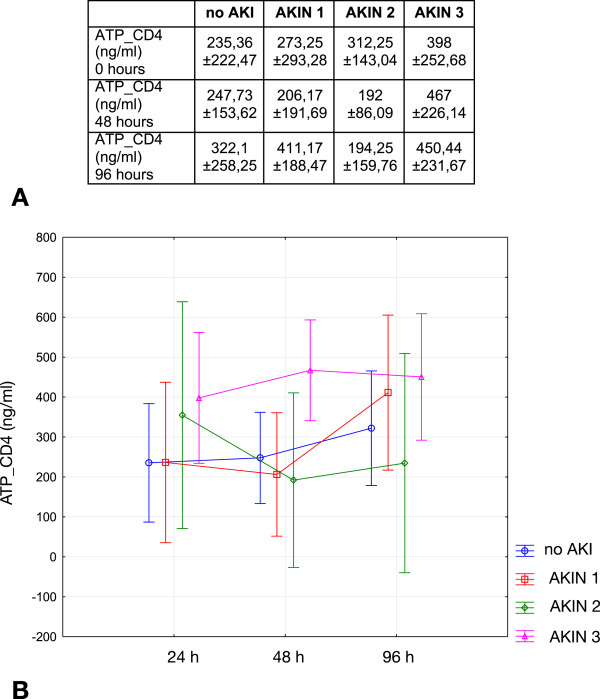
**ATP_CD4 at different time points.** Contents did not differ between the four groups and also not between the time points analyzed. **A** shows the numerical results, **B** summarizes dynamics of ATP_CD4 over time (Data as mean ± SD).

**Figure 2:**
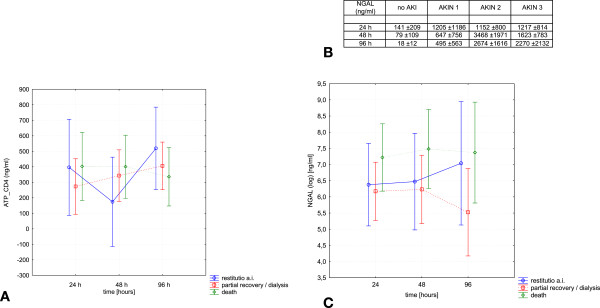
**A - ATP_CD4 related to the three categories complete renal recovery, partial renal recovery/persistent need for dialysis, and sepsis-associated death.** At 48 h, patients with complete renal recovery tended to have lower concentrations of ATP_CD4 compared to the two other groups. **B** and **C** - Urinary NGAL in all patients of the four groups **(B)** and **(C)** related to the categories complete renal recovery, partial renal recovery / persistent need for dialysis, and sepsis-associated death. NGAL gradually increased with increasing severity of AKI **(B)**. Differences were not significant if related to the prognosis **(C)** (Data as mean ± SD).

Primary study endpoint was ATP content in CD4+ T cells. This parameter was analyzed in both a longitudinal (between groups - no AKI, AKIN 1-3) and a horizontal (comparison at different time points - 24, 48, and 96 h) manner. Differences between the groups were not significant (p = 0.15), those between the three time points were also not different (p = 0.11). The results including ATP concentrations are summarized in Figure 1. ATP_CD4 was then evaluated in relation to the following parameters: death, partial renal recovery/dialysis, complete renal recovery. Analyses were once again performed in a longitudinal and horizontal manner. Differences were not statistically significant between any of the three groups at any of the time points analyzed. However, patients with complete recovery of renal function tended to have lower concentrations of ATP_CD4 at 48 hours as compared to patients with partial recovery or death (Figure 2). An additional performed power analysis, using the programme G*Power® showed that a minimum of 159 patients would have to be included into the study in order to prove suspected significance (53 individuals per group - death, partial, complete renal recovery).

### Secondary study endpoint: urinary NGAL

Urinary NGAL has been shown to serve as prognostic marker in AKI [2]. Thus, it is somehow an established parameter of acute kidney dysfunction. We therefore measured its concentration in each group/time point, and also related it to the three categories - death, partial renal recovery/dialysis, complete renal recovery - in order to draw more sophisticated conclusions about ATP_CD4. We identified that urinary NGAL gradually increased with progressive renal damage in AKI. The lowest values were measured in patients with no AKI at all (Figure 2). Patients with AKI and death tended to show higher NGAL concentrations than patients with complete or partial renal recovery/dialysis. The latter results were however not statistically significant. A detailed summary of the results is given in Figure 2.

## Discussion

Our investigation shows that ATP_CD4 may potentially serve as marker to discriminate between patients with better post-AKI outcome (complete renal recovery) from patients with partial recovery of renal function including persistent need for dialysis, and from patients with sepsis-associated death. At 48 hours after inclusion into the study, ATP_CD4 tended to be lower in concentration in comparison to the two other groups. Nevertheless, differences were only close to the level of significance, most likely as a result of the limited number of subjects. We extrapolated the number of patients necessary for detecting a significant difference. A minimum total number of 159 individuals with 53 subjects per individual group would have to be included into the investigation. Such expanded study is being initiated at the moment.

Urinary NGAL was useful for differentiating the severity of acute renal dysfunction. It gradually increased with progression of AKI from stage 1 to 3 according to the AKIN criteria. It still remains a fundamental goal to identify new marker molecules of acute renal damage, as they are useful for diagnosing AKI at an early stage, and should also aid with the prediction of both renal and overall outcome of patients with AKI. Serum creatinine has been used for more than 50 years now [14]. Although a number of new promising candidates were identified in recent years, including Cystatin C [13, 15], NGAL [16], KIM-1 [17], none of them have shown to be superior to the others in terms of early AKI detection. In addition, predicting the *prognosis* of AKI is still very difficult. This results from the fact that most of the diagnostic markers available today are *eliminated* by the kidney, but do not indicate processes involved in tissue regeneration and repair. Septic AKI significantly results from renal hypoperfusion causing damage of the tubular epithelium. Other consequences include intrarenal inflammation and peritubular microvasculopathy; both significantly modify dynamics of postischemic tissue regeneration [5]. Nevertheless, indirect methods for monitoring tissue damage and repair are still lacking. Postischemic intrarenal inflammation is initiated by ischemia-induced release of proinflammatory cytokines by tubular epithelial and vascular endothelial cells, respectively [18]. Increased IL-6 serum levels have been shown to predict mortality in AKI [19]. In order to monitor activity of T cells in sepsis, a larger cohort of sepsis patients was analyzed for ATP concentrations in the cells (ATP_CD4) [5]. The investigation showed higher ATP_CD4 in survivors as compared to non-survivors. Although one may conclude that higher ATP_CD4 indicate increased cell competence, it is still not possible to draw any definite conclusions about the relevance of this finding in the process of self-repair. Our study, on the other hand, revealed lower ATP_CD4 levels in patients with complete renal recovery after sepsis-associated AKI. This observation is in line with more recent data about ATP_CD4 in renal transplant recipients. In this particular study, increased ATP levels predicted acute renal allograft rejection [20]. Therefore, lower ATP_CD4 levels may indicate a lower risk for immune-mediated kidney damage ultimately promoting faster recovery from sepsis-associated AKI. However, at this point it is too early to establish ATP_CD4 as new diagnostic parameter in sepsis-associated AKI or even to draw definite conclusions about its role in AKI risk prediction. Further studies must be conducted to confirm the promising results of the current investigation. Particular interest will be related to additional time points of ATP_CD4 analysis. It needs to be determined whether ATP_CD4 concentration changes very early, for instance before sepsis can even be diagnosed. Thus, patients with increased risk for developing sepsis (e.g. chemotherapy, other immunosuppressive drugs, diabetes with severe non-septic infection) will be monitored as well. It should also be mentioned that the chronic uremic milieu in CKD may modulate ATP_CD4 in a way that dynamic alterations of ATP content cannot truly be compared with alterations seen in patients without preexisting CKD. That aspect would also have to be considered in further studies. Finally, a more detailed analysis of plasma NGAL levels is also needed in order to helpidentify patients with increased risk for CKD after AKI.

## Conclusion

In conclusion, ATP_CD4 is a promising candidate for predicting renal prognosis in sepsis-associated AKI. Lower concentrations at 48 hours after onset of the disease may indicate a higher chance for complete renal recovery. Further investigations are needed, and are underway, in order to specify its role in AKI risk prediction.

## Abbreviations

AKI: Acute kidney injury
AKIN: Acute kidney injury network
ATP_CD4: ATP content of CD4+ t cells
CKD: Chronic kidney disease
KIM-1: Kidney injury molecule-1
NGAL: Neutrophil gelatinase-associated lipocalin
SIRS: Systemic inflammatory response syndrome.
